# Methane and ethane detection from natural gas level down to trace concentrations using a compact mid-IR LITES sensor based on univariate calibration

**DOI:** 10.1016/j.pacs.2023.100448

**Published:** 2023-01-04

**Authors:** Andrea Zifarelli, Angelo Sampaolo, Pietro Patimisco, Marilena Giglio, Miguel Gonzalez, Hongpeng Wu, Lei Dong, Vincenzo Spagnolo

**Affiliations:** a State Key Laboratory of Quantum Optics and Quantum Optics Devices, Institute of Laser Spectroscopy, Shanxi University, Taiyuan 030006, China; b PolySense Lab, Dipartimento Interateneo di Fisica, University and Politecnico of Bari, via Amendola 173, Bari, Italy; c PolySense Innovations Srl, via Amendola 173, Bari, Italy; d Aramco Services Company, Aramco Research Center, Houston, United States

**Keywords:** LITES, Methane, Ethane, Multi-gas sensing, Natural gas, Environmental monitoring

## Abstract

A gas sensor based on light-induced thermo-elastic spectroscopy (LITES) capable to detect methane (C1) and ethane (C2) in a wide concentration range, from percent down to part-per-billion (ppb), is here reported. A novel approach has been implemented, exploiting a compact sensor design that accommodates both a custom 9.8 kHz quartz tuning fork (QTF) used as photodetector and the gas sample in the same housing. The resulting optical pathlength was only 2.5 cm. An interband cascade laser (ICL) with emission wavelength of 3.345 µm was used to target absorption features of C1 and C2. The effects of high concentration analytes on sensor response were firstly investigated. C1 concentration varied from 1% to 10%, while C2 concentration varied from 0.1% to 1%. These ranges were selected to retrace the typical natural gas composition in a 1:10 nitrogen dilution. The LITES sensor was calibrated for both the gas species independently and returned nonlinear but monotonic responses for the two analytes. These univariate calibrations were used to retrieve the composition of C1-C2 binary mixtures with accuracy higher than 98%, without the need for further data analysis. Minimum detection limits of ∼650 ppb and ∼90 ppb were achieved at 10 s of integration time for C1 and C2, respectively, demonstrating the capability of the developed LITES sensor to operate with concentration ranges spanning over 6 orders of magnitude.

## 1 Introduction

In the last years, the development of reliable gas sensing technology has rapidly become a mandatory task for several crucial applications, from environmental monitoring to human health care [1], [2], [3], [4]. Among the others, hydrocarbons detection has gained importance throughout the years in different applications as environmental monitoring, energy transition, and natural gas (NG) analysis [5]. NG is the generic term used to indicate mixtures of hydrocarbon and nonhydrocarbon compounds commonly associated with petroliferous and geologic formations, as well as biological sources (biogas). Typical NG composition accounts for a predominant share of methane (C1), more than 70%, and smaller fractions of heavier hydrocarbons as ethane (C2), propane (C3), butane (C4) as well as nonhydrocarbon species (CO_2_, H_2_S, N_2_.) in the percentage range [6], [7]. NG represents one of the most important energetic sources worldwide, whose interest is rising due to its role in decarbonization and energetic transition towards green hydrogen [8]. These applications need different specifications and regulations, thus it is crucial to have a reliable monitoring on NG samples in all their life cycle: production, treating, transportation, processing and final use [9]. The standard approach for NG analysis relies on analytical methods, based on gas chromatography, but these approaches are typically laboratory-based and characterized by a large footprint [10]. Therefore, based on the currently available analysis tools, a real-time and in situ monitoring of the gaseous samples’ composition for on-field applications (e.g., leak detection) is not of straightforward implementation [11]. In this scenario, gas sensors based on infrared spectroscopy have proved to represent as a solid alternative to analytical approaches, being characterized by high sensitivity, small footprint, and fast response time [12]. Several gas sensors for hydrocarbons detection were developed employing optical-based detection techniques. These sensors were mainly operate for methane and ethane detection in trace concentrations for environmental monitoring purposes, exploiting spectroscopic methods as cavity-based techniques [13], [14], non-dispersive infrared sensors [15], [16], photoacoustic spectroscopy [17], [18], [19], photothermal spectroscopy [20], [21], and tunable diode laser absorption spectroscopy (TDLAS) [22], [23]. These optical sensors exploit the absorption features located in the near- and mid-IR and corresponding to the C-H bond stretching, which characterize the absorption spectra of several alkanes and hydrocarbons (fundamental mode lies in λ = 3–4 µm, first overtone in λ = 1–2 µm). The increasing interest for NG analysis has led to the development of laser-based sensors capable of simultaneous detection of multiple hydrocarbons but also capable to cover a concentration range reaching up to percent [24], [25], [26], [27]. However, the extension towards high concentrations it is not granted for free. In fact, when moving towards natural gas-like concentrations, the response and selectivity of an optical sensor might be affected by non-linear absorption and spectral interference, respectively. For some spectroscopic configurations, such as the photoacoustic technique, non-spectral interference (i.e., cross-sensitivity effects) arises also as a potential further detrimental factor [28]. Therefore, statistical tools based on multivariate analysis were applied to the above-mentioned sensors to retrieve analytes concentrations [29]. With the aim of reducing the impact of the spectral and non-spectral interference, an optical gas sensor should be in principle designed to target well separated absorption features of the target hydrocarbons. In addition, the sensing system should be conceived to avoid any cross-sensitivity among the gas species, even at high concentrations. This would be the optimum scenario to develop a versatile gas sensor suitable for multiple on-field detection of hydrocarbons, whose concentrations can span from typical NG concentration levels down to traces dispersed into environment. Light-induced thermo-elastic spectroscopy (LITES) is a recent development of traditional TDLAS scheme [30]. In this configuration, gas detection is typically performed by means of a wavelength modulation approach (WM) [31], employing a quartz tuning fork (QTF) as light detector [32]. In LITES sensors, the laser beam passes through an absorption cell containing the target gas samples and is modulated at the resonance frequency of the QTF or its subharmonics. Then, the beam spot is focused on the QTF surface giving rise to a local heating: the thermal energy is then converted into elastic deformation, i.e., strain field, generating in turn electric charges due to the piezoelectric properties of quartz [33]. The architectures of LITES sensors reported to date rely on a gas absorption cell and the QTF-based detector placed at the cell exit [34], [35], [36]. In this configuration, several LITES sensors were developed to detect trace concentrations of C1 [37], [38]. Nevertheless, neither detection of heavier hydrocarbons nor investigations at higher concentrations have been investigated to date.

In this work, a novel LITES sensor for simultaneous detection of C1 and C2 in a wide range of concentrations is proposed. These molecules were selected among the other hydrocarbons due to their relevance in the evaluation of NG origin and reservoir composition [39], as well as their crucial role in NG handling and transportation [40]. With aim of increasing the detectable concentration range while guaranteeing a sub-ppm sensitivity and reducing at the same time the footprint of LITES configurations, differently from the configuration employed up to now, the sensor architecture was re-designed to enclose both the gas sample and the QTF detector within the same compact cell. The proposed design is similar in size to the acoustic detection module employed in a quartz-enhanced photoacoustic spectroscopy sensors [27], resulting in an absorption pathlength of few centimeters. The small-size sensor was capable to retrieve mixture compositions by means of simple univariate calibration, thus being suitable for real-time, in situ monitoring of C1 and C2. Moving from high to low analytes concentrations, the detection limits of the proposed configuration were investigated by exploiting the QTF response dependence on the beam spot position over the QTF surface.

## 2 Experimental setup

**Fig. 1:**
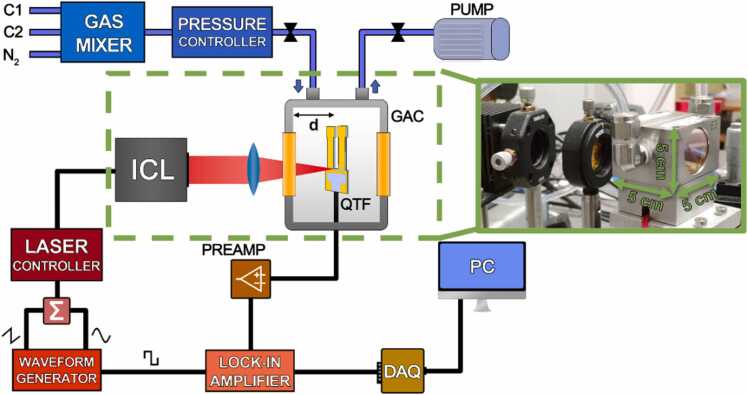
Experimental setup. C1, methane; C2, ethane; ICL, interband cascade laser; QTF, quartz tuning fork; GAC, gas absorption cell; DAQ, digital acquisition; PC, personal computer; Σ: adder; PREMAP: transimpedance preamplifier. (inset) Core of the realized GAC. The dimensions of the GAC are shown in the photo.

A schematic representation of the experimental setup employed in this work is shown in Fig. 1.

The laser source used to target the absorption features of both C1 and C2 was a distributed-feedback interband cascade laser (DFB-ICL) manufactured by Nanoplus GmbH. The device was operated at T = 15 °C, providing a maximum output power of ∼9 mW and a central emission wavelength of 3.345 µm (2989.5 cm^−1^). This spectral range was selected as it provides well separated absorption features of C1 and C2 [27], [41]. The ICL was controlled by means of a combined laser driver and TEC controller (Thorlabs ITC 4002) without the need for an external cooling. An infrared power meter was used for alignment purposes (Thorlabs S401C, not shown in Fig. 1). The ICL beam was focused on the QTF surface using a 2–5 µm AR-Coated silicon lens with focal length *f* = 50.0 mm (Thorlabs LA8862-E). The beam spot size was measured at the lens focal plane using a pyrocamera (Spiricon IIIHR), and a circular profile with a full width at half maximum (FWHM) of 300 µm was observed.

The tuning fork employed as photodetector was a custom T-shaped QTF with 50 µm-deep rectangular grooves carved on both prong sides, which had already demonstrated to provide the best performances in LITES experiments [32]. The QTF was housed inside a custom-made gas absorption cell (GAC), a stainless-steel vacuum-tight cell equipped with two wedged BaF_2_ windows (Thorlabs WW01050-E1, AR Coating: 2–5 µm). As shown in Fig. 1, the optical absorption path available for the gas detection was limited to the space between the first window of the GAC and the QTF, i.e., *d* = 2.5 cm. The GAC consisted in a metal cube of 5 cm side, as shown in the inset of Fig. 1.

The sensor was operated in *2 f*-WM, as the laser current was modulated at half of the QTF resonance frequency by means of a waveform generator (Tektronix AFG 3102). The sine wave modulation was superimposed to a slow triangular ramp, used to sweep the laser injection current and investigate the whole laser dynamic range. The QTF piezoelectric current generated by the thermoelastic conversion of the modulated laser light was collected and transduced into a voltage signal by means of a custom transimpedance preamplifier [42]. The modulated voltage signal was collected by a lock-in amplifier (EG&G model 7265) to be demodulated at an integration time of 100 ms. The lock-in output signal was then sent to a DAQ card (National Instrument USB-6356-BNC) and recorded and visualized on a PC using a custom LabVIEW-based software.

Gas cylinders with certified concentrations were used to generate mixtures with different concentrations of C1 and C2. Following the same procedure employed in Ref. [27], the investigation upon natural gas-like samples was carried out by mimic a 1:10 safety dilution of typical NG concentrations. Therefore, to investigate the high concentrations range two cylinders containing 10% C1:N_2_ and 1% C2:N_2_ were employed. Conversely, the investigation upon the sensors minimum detection limits was performed using two gas cylinders with certified mixtures 1% C1:N_2_, and 0.1% C2:N_2_. The gas concentration in the certified cylinders is provided with a 4% expanded uncertainty. Pure N_2_ was employed to dilute the certified mixtures. A gas mixer (MCQ Instruments GB-100) was used to set both the C1-C2-N_2_ mixing ratio in the samples and the flow rate within the gas line. All the measurements were acquired in continuous flow regime at 50 sccm. The sensor’s operating pressure was set to atmospheric level (P = 760 Torr) using a pressure controller (MKS Type 649) while the steady flow regime was provided by a rotative pump (KNF N813).

## 3 Quartz tuning fork characterization in different surrounding environments

The developed LITES sensor is characterized by the coexistence of the target gases and the detector in the same cell. The main drawback is that both the QTF frequency (*f*) and *Q*-factor could be influenced by the surrounding environment composition [43]. Indeed, in LITES detection, the QTF response depends on the product between the QTF accumulation time $\tau =\frac{Q}{2\pi f}$ and the strain field intensity (ε) generated on the area where the laser beam is focused. Therefore, aiming to explore a large concentration range for both C1 and C2, the effects of sample composition on the QTF response was primarily evaluated. The main loss mechanism affecting a vibrating QTF are support losses, thermoelastic losses, and viscous losses. The latter are caused by the drag force exerted on the QTF when immersed in a viscous medium, i.e., the gas sample [43]. Aiming to investigate the effects of different gas matrices respect to QTF response, both support losses and thermoelastic losses can be assumed constant, while viscous losses depend on the target gas samples. A theoretical approximation of the Q-factor related to fluid damping (*Q*_*gas*_) for a cantilever beam was provided by Hosaka et al. [44]:

(1) $$Q_{\mathrm{gas}=}\frac{4\rho TW^{2}f_{n}}{3\mu_{\mathrm{gas}}W+\frac{3}{4}W^{2}\sqrt{\left(4\pi \rho_{\mathrm{gas}}\mu_{\mathrm{gas}}f_{n}\right)}}$$

where *ρ* is the quartz density, *T* is the thickness of the quartz crystal, *W* is the width of rectangular QTF prong, *f*_*n*_ is the QTF resonance frequency, *ρ*_*gas*_ is the density of the gas matrix and µ_*gas*_ is the gas matrix dynamic viscosity. For a gas mixture, µ_*gas*_ can be estimated according to the formula [45]:

(2) $$\mu_{gas}=\frac{\Sigma \;\mu c_{i}\sqrt{M_{i}}}{\Sigma \;c_{i}\sqrt{M_{i}}}$$

where the subscript *i* indicates the gas species, *c* the gas concentration and *M* the molecular weight. Both *M* and *ρ*_*gas*_ can be calculated as an average weighted on components’ concentrations. The effects of the surrounding medium on the QTF fundamental resonance frequency can be modelled as [46]:

(3) $$f=f_{\mathrm{vac}}\left(1-\frac{u*P}{2\rho_{\mathrm{gas}}\mathrm{WT}}\right)$$

where *f*_*vac*_ is the resonant frequency in vacuum and *u* is the added mass generated by the fluid-structure interaction. The latter parameter depends on the cantilever geometry as well as the fluid properties as density, hydrodynamic function, and Reynold’s number [47].

As an example, the *Q*_*gas*_ contribution related to four representative gas mixtures were calculated using (1), (2). The selected mixtures compositions were: pure N_2_, 9% C1 in N_2_, 0.5% C2 in N_2_, and 9% C1 + 0.5% C2 in N_2_. The fluid damping losses were calculated assuming a QTF with rectangular prongs characterized by *T* = 250 µm, *W* = 1.4 mm, *ρ* = 2650 kg/m^3^, and *f*_*n*_ = 9792.6 Hz. The collected results as well as the parameters employed for calculations [48] are reported in Table 1.

**Table 1:** Theoretical Q_gas_ calculation: fluid dynamics parameters and corresponding results for the selected gas mixtures.

Mixture	ρ_gas_ [kg/m^3^]	µ_gas_ [Pa∙s]	M [kg/mol]	Q_gas_
Pure N_2_	1.132	18.50 ∙10^−6^	28.01 ∙10^−3^	1.019 ∙10^5^
9% C1:N_2_	1.089	17.83 ∙10^−6^	26.94 ∙10^−3^	1.058 ∙10^5^
0.5% C2:N_2_	1.132	18.45 ∙10^−6^	28.02 ∙10^−3^	1.020 ∙10^5^
9% C1 + 0.5% C2	1.090	17.79 ∙10^−6^	26.95 ∙10^−3^	1.059 ∙10^5^

**Fig. 2:**
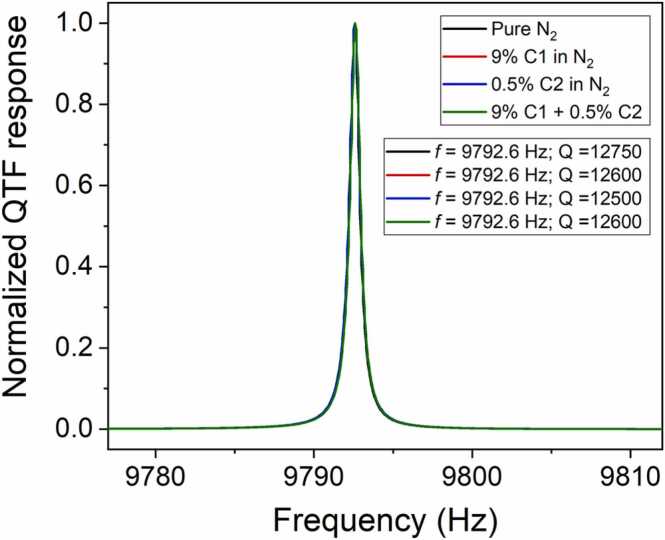
QTF electric response collected at atmospheric pressure for different gas matrices: pure N_2_ (black curve), 9% C1 in N_2_ (red curve), 0.5% C2 in N_2_ (blue curve), 9% C1 and 0.5% C2 in N_2_ (green curve). Calculated values for resonance frequencies and quality factors are reported in the legend.

A maximum percent variation of ∼3.5% was calculated among the theoretical fluid damping losses corresponding to the analyzed samples. The theoretical results were compared to the electrical characterization of the QTF employed in the LITES sensor performed in the above-mentioned gas mixtures. The experimental results are reported in Fig. 2.

The resonance frequency and the quality factor measured in pure N_2_ environment at atmospheric pressure (black curve in Fig. 2) were *f*_*atm,N2*_ = 9792.6 Hz and *Q*_*atm,N2*_ = 12,750. No significant effects of the gas matrices on QTF response were observed in the investigated concentration range, as the retrieved resonance frequency was equal to 9792.6 Hz for all the mixtures and the measured Q-factor variations were below 3%. As a result, the QTF response when changing the mixture composition was considered flat and no additional scalings were applied.

## 4 High-concentration hydrocarbons detection

With respect to trace-gas analysis, the detection of analytes at high concentrations could provide high voltage signals in turn, thus it is crucial to avoid saturation of the electrical response of the instrumentation employed. Using the QTF as photodetector, it is possible to vary the excited strain field intensity by changing the laser beam focusing position [32], thus keeping the voltage signals below a threshold value. This is similar to select a given gain value on a commercial photodetector for avoiding the saturation of the output electric signal [49]. Since the lock-in amplifier employed in the experimental setup was characterized by a maximum input voltage of 1 V, the position of the laser beam on QTF surface was preliminarily optimized to guarantee an output voltage signal not exceeding the operating range of the instrument. This position was labelled as low sensitivity position (LSP). Then, the LITES sensor was calibrated by detecting C1 and C2 at different concentration levels, in nitrogen matrix.

**Fig. 3:**
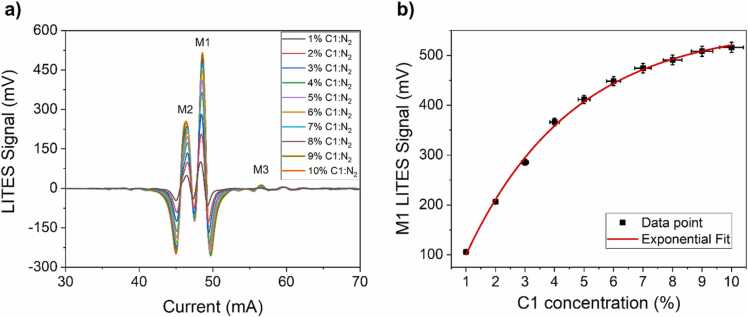
(a) 2 f-spectral scan of C1 absorption lines at concentration levels varying from 1% up to 10% in N_2_ at a pressure of 760 Torr. The most distinguishable absorption features are labelled as M1, M2 and M3, respectively. (b) Peak signal of the absorption feature M1 as a function of C1 concentration (black squares) and corresponding exponential fit (red line).

Ten different dilutions of C1:N_2_ were generated using the gas mixer, and *2f*-QEPAS spectral scans were acquired by sweeping the ICL injection current from 30 mA to 70 mA. The collected spectra are shown in Fig. 3a. All the measurements reported hereafter were acquired by optimizing both the lock-in detection phase and the sinusoidal modulation amplitude for C1 detection.

Three main features can be recognized from the C1 spectral scan, labelled as M1, M2 and M3. Following the HITRAN database [50], peak M1 corresponds to an absorption line of methane located at 2988.795 cm^−1^ with an absorption linestrength of 1.075∙10^−19^ cm/mol. Peak M2 corresponds to two absorption lines, located at 2988.932 cm^−1^ and 2989.033 cm^−1^, which are merged because of pressure broadening. Peak M3 corresponds to an absorption line located at 2987.882 cm^−1^ and characterized by an absorption linestrength of 6.951∙10^−22^ cm/mol, much weaker than M1 and M2. The peak signals of the absorption features were extracted, and the LITES signal of peak M1 as a function of C1 concentration is reported in Fig. 3b. The error bars on the Y-axis correspond to the measured signal amplitude fluctuation of 2%, while the error bars on the X-axis correspond to the 4% expanded uncertainty associated to analyte concentration in the cylinder. Peak M1 follows the Lambert-Beer law for non-weak absorptions. To retrieve a calibration curve for the sensor, an exponential fit was performed to interpolate the peak values using the following fitting function:

(4) $$y=y_{0}-A∙e^{{-R}_{0}∙x}$$

The retrieved best fit parameters were y_0_ = 558 ± 10 mV, A = 604 ± 10 mV and R_0_ = 0.28 ± 0.02.

**Fig. 4:**
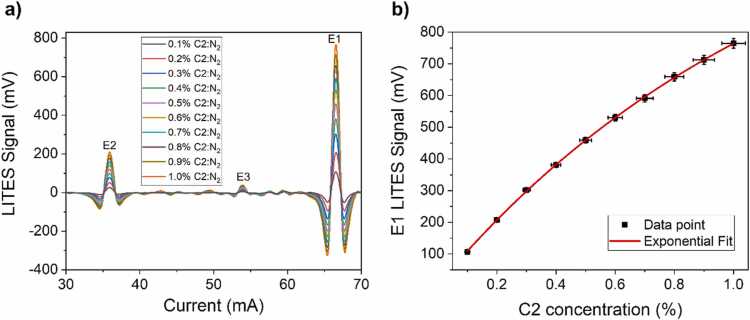
(a) 2 f-spectral scan of C2 absorption lines at concentration levels varying from 0.1% up to 1.0% in N_2_ at a pressure of 760 Torr. The most distinguishable absorption features are labelled as E1, E2 and E3, respectively. (b) Peak signal of the absorption feature E1 as a function of C2 concentration (black squares) and corresponding exponential fit (red line).

In order to calibrate the LITES sensor for C2 detection, ten different dilutions in nitrogen were generated. Analogously to C1 detection, the *2 f*-QEPAS spectral scans of C2 mixtures were acquired by tuning the ICL injection current from 30 mA to 70 mA. The acquired spectra are displayed in Fig. 4a.

C2 spectral scans point out three well-recognizable absorption features, labelled as E1, E2 and E3. Peak E1 corresponds to an absorption band of ethane located in the spectral interval from 2986.574 cm^−1^ to 2986.729 cm^−1^. The most intense absorption line in the band is characterized by an absorption linestrength of 3.21∙10^−20^ cm/mol. Similarly, peak E2 corresponds to an ethane absorption band, located in the spectral interval from 2989.923 cm^−1^ to 2990.095 cm^−1^. Peak E3 is the result of the merging of multiple absorption line located around 2988.1 cm^−1^. The peak signals of all these features were extracted, and the LITES signal of peak E1 as a function of C1 concentration is reported in Fig. 4b. As for peak M1, the peak values of E1 were fitted using Eq. 3 and the results are shown in Fig. 4b. The retrieved best fit parameters were y_0_ = 1376 ± 36, A = 1374 ± 33 and R_0_ = 0.81 ± 0.03.

**Fig. 5:**
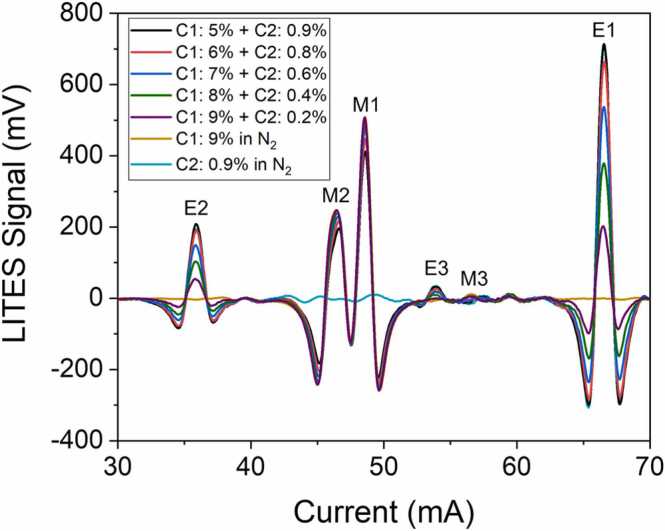
2f-spectral scans of five mixtures containing 5% of C1 and 0.9% of C2 (black curve), 6% of C1 and 0.8% of C2 (red curve), 7% of C1 and 0.0.6% of C2 (blue curve), 8% of C1 and 0.4% of C2 (green curve), 9% of C1 and 0.2% of C2 (purple curve), all in N_2_. 2f-spectral scans measured for mixing containing 9% of C1 in N_2_ (gold curve) and 0.2% of C2 in N_2_ (azure curve) are also shown for comparison.

With the aim of assessing the LITES signal behavior when both C1 and C2 are flushed through the sensor, 25 mixtures were generated using the gas blender. The analytes concentrations were selected to simulate their typical ratios within a natural gas composition [6] 1:10 diluted in N_2_, i.e., C1 from 5% to 9% and C2 from 0.2% to 0.9%. The *2f*-QEPAS spectral scans across the ICL tuning range were performed in the same operating conditions employed for the single-species calibration. The spectra obtained from five C1-C2 mixtures are shown in Fig. 5 as representatives, together with two spectra related to samples containing only C1 and C2, respectively.

**Fig. 6:**
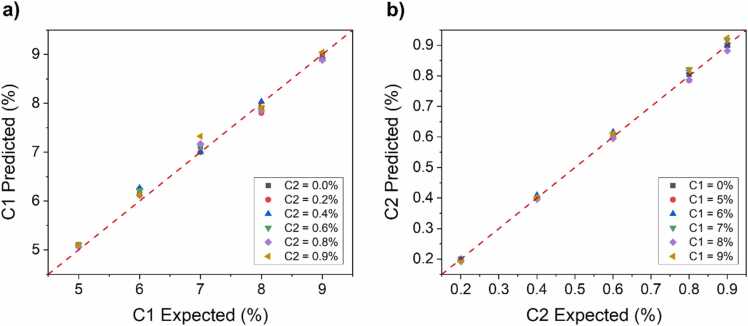
a) C1 predicted concentrations versus expected concentrations. b) C2 predicted concentrations versus expected concentrations. Red dashed line is a guide to the eye.

Negligible interference was observed among the most intense spectral features of both C1 and C2 spectra, i.e., M1, M2, E1, and E2, as already verified by several works in literature [27], [41]. LITES signals comparable to noise level were collected at I = 48.5 mA (peak M1) as well as at I = 66.5 mA (peak M2) when only C2 or only C1 were present in the gas sample, respectively. Conversely, a moderate spectral interference among M3 and E3 can be observed. Therefore, the concentrations of the analytes in mixtures can be retrieved using the sensor’s calibration curves shown in Fig. 3b and Fig. 4b for C1 and C2, respectively. The calculated concentrations compared to the expected ones are plotted in Fig. 6a and Fig. 6b as datapoints.

Red dashed lines are guides to the eye representing the perfect match among predicted and expected concentrations. The average relative accuracy error on the retrieved concentrations was equal to 2.6% and 1.6%, with a maximum relative deviation of 5.8% and 4.3% for C1 and C2, respectively.

**Fig. 7:**
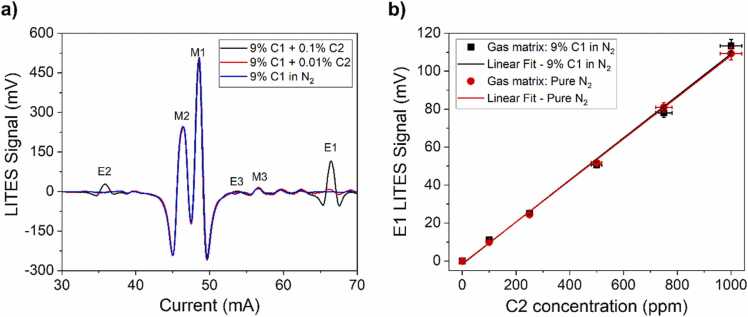
a) 2 f-spectral scans of two gas mixtures containing 9% of C1 and 0.1% (1000 ppm) of C2 (black curve), 9% of C1 and 0.01% (100 ppm) of C2 (red curve), and 9% of C1 (blue curve), respectively, in N_2_. b) E1 peak signal as a function of C2 concentration measured in pure N_2_ matrix (red dots) and corresponding best linear fit (red solid line). E1 peak signal as a function of C2 concentration measured in a 9% C1:N_2_ matrix (black squares) and corresponding best linear fit (black solid line).

The negligible interference among the analytes’ absorption features in this spectral range allows the sensor to be tested on gas mixtures characterized by large mixing ratios. The simultaneous evaluation of both C1 and heavier hydrocarbons, such as C2, at lower concentration is a valuable method used to distinguishing different methane sources (biogenic, thermogenic) [39]. With this aim, several mixtures composed of a fixed concentration of 9% C1:N_2_ and a variable concentration of C2 in the ppm range were investigated. For these measurements, C2 was provided by a gas cylinder with certified concentration of 1000 ppm (0.1%) C2:N_2_ to be diluted in nitrogen down to 100 ppm (0.01%). In Fig. 7a the spectral scans of two representative mixtures are reported, as well as the spectral scan acquired for 9% C1:N_2_.

Ethane absorption peak E1 was clearly distinguishable at an injection current of 66.5 mA, and no interference from C1 absorption background was observed. E1 peak values were extracted and are reported in Fig. 7b as a function of C2 concentration in mixture. The experimental points corresponding to no C2 in mixture (0 ppm) were collected by flushing a pure N_2_ and a 9% C1:N_2_ mixture. No significant difference in terms of acquired signal was observed in C2 detection channel for the two gas samples without ethane. The LITES signals at different concentrations exhibited a linear trend in the investigated concentration range, consistently with the decreasing absorption strength and in accordance with the measurements shown in Fig. 4b. A linear fit was performed on E1 peak values extracted from both N_2_-based matrix and C1 9%:N_2_-based matrix, returning a slope of 0.110 ± 0.002 mV/ppm (R^2^ = 0.9991) and 0.111 ± 0.004 mV/ppm (R^2^ = 0.995). Both the linear fits showed a negligible intercept within the error limits.

## 5 Trace-gas hydrocarbons detection

**Fig. 8:**
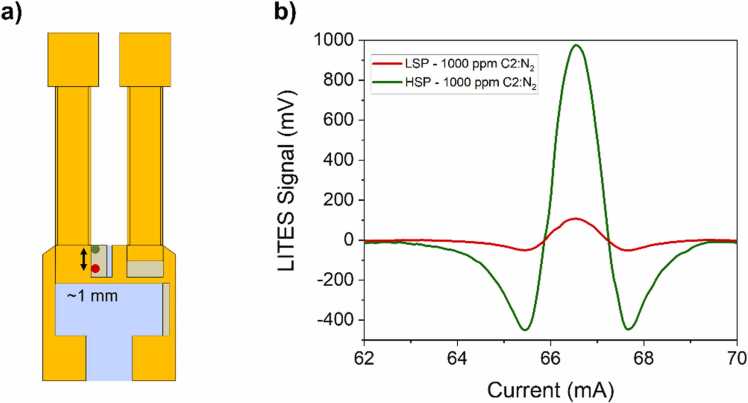
a) Schematic of the QTF geometry. LSP is shown as red dot while HSP is shown as green dot, on QTF surface. b) LITES spectral scans of peak E1 for a 1000 ppm C2:N_2_ gas mixture acquired targeting the LSP (red curve) and the HSP (green curve).

The sensing capabilities of the sensor prototype were also tested down to the minimum detection limits (MDLs) for both the analytes, in order to evaluate the effective dynamic range in concentration. Aiming at enhancing the QTF response and increase the sensor’s sensitivity, the laser beam position on the QTF surface was adjusted to achieve the maximum LITES signal. Therefore, the beam spot was moved from the LSP and focused on the area providing the maximum strain field, close to the prong’s base, as shown in Fig. 8a. This position was labelled as high sensitivity position (HSP). As a preliminary investigation, the LITES signal enhancement when moving from LSP to HSP was estimated. The LITES spectral scans across peak E1 acquired while flowing a gas sample containing 1000 ppm of C2:N_2_ and targeting both LSP and HSP are shown in Fig. 8b.

**Fig. 9:**
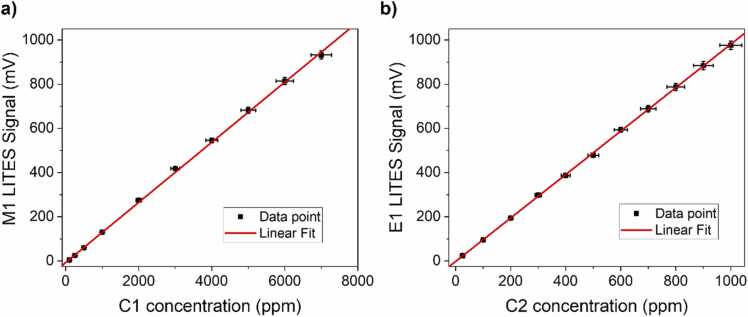
(a) LITES signal of peak M1 as a function of C1 concentration (black squares) and corresponding best linear fit (red line). (b) LITES signal of peak E1 as a function of C2 concentration (black squares) and corresponding best linear fit (red line).

Moving from LSP to HSP, the recorded peak signal increased from 105 mV to 975 mV, thus resulting in a signal enhancement of ∼9.3 times. To generate mixtures with low analytes concentrations, a gas cylinder with a certified concentration of 1% C1:N_2_ and a gas cylinder with a certified concentration of 1000 ppm C2:N_2_, already employed in the previous section, were used. Both the gas cylinders were provided with an expanded uncertainty of relative 4%. These concentrations match the typical range observed for NG leakage from pipelines [51]. The target gases were independently diluted in N_2_ and the LITES signals corresponding to the peaks M1 and E1 were measured. Compared to the measurements performed at high concentration, no changes to sensor’s parameters (operating pressure and temperature, flow rate) were made when targeting low concentrations. The collected results are shown in Fig. 9.

**Fig. 10:**
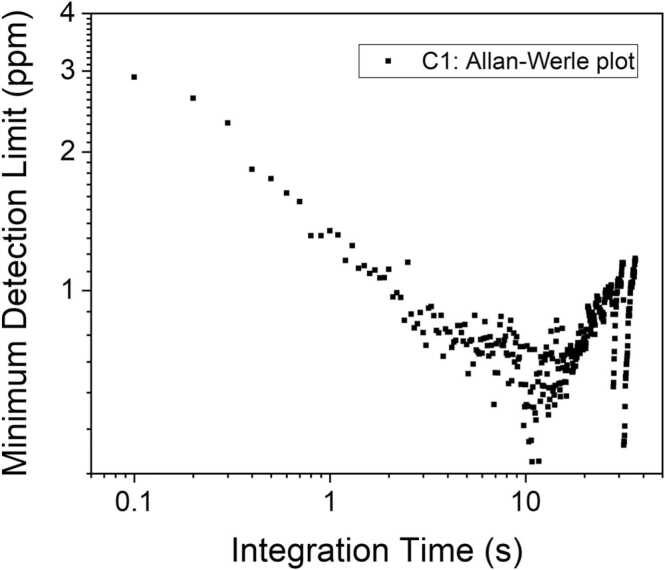
Plot of C1 minimum detection limit as a function of integration time, calculated as result of Allan-Werle deviation analysis.

The peak signals extracted for both M1 and E1 pointed out a perfectly linear trend with respect to the analytes’ concentrations, as demonstrated by the linear fit superimposed to the data (red line in Fig. 9). The fitting procedure returned a slope of 0.136 ± 0.001 mV/ppm (R^2^ = 0.9993) and 0.983 ± 0.005 mV/ppm (R^2^ = 0.9997) mV/ppm for C1 and C2, respectively, with negligible intercept within the error limits. Being verified the linear response of the LITES sensor for analytes at low concentrations, the minimum detection limits (MDLs) were estimated as the concentrations corresponding to SNR = 1. The 1σ-noise measured when setting an integration time of 100 ms at M1 peak current was 0.405 mV, while the corresponding 1σ-noise measured at the E1 peak current was 0.370 mV. Therefore, the calculated MDLs were 2.9 ppm and 375 ppb for C1 and C2, respectively, at 100 ms of integration time. To test the stability of the developed sensor and estimate the ultimate detection limits, an Allan-Werle deviation analysis was performed by flowing pure nitrogen in the ADM and acquiring the LITES signal operating the ICL at the injection currents corresponding to peaks M1 and E1. The results of Allan-Werle analysis for C1 are shown in Fig. 10 as representatives.

From the Allan-Werle analysis, MDLs of 1.3 ppm and 170 ppb were estimated at 1 s of integration time for C1 and C2, respectively. Increasing the integration time up to 10 s, MDLs of ∼650 ppb and ∼90 ppb were reached. Longer integration times lead to the decrease of sensor performances, because of mechanical instabilities.

## 6 Conclusions

In this work, a novel optical gas sensor based on LITES was proposed to address the main requirements of real-time and in situ gas sensing application characterized by multicomponent samples: wide operative concentration range, high sensitivity, low degree of cross-correlations and low interference among the target analytes, high level of compactness and ruggedness. This LITES sensor was developed for hydrocarbon detection in particular to set a direct comparison with previously demonstrated C1/C2 QEPAS, characterized by sophisticated multivariate approaches to level off the influence of the fluctuating gas matrices [27]. The novel sensor design was based on a single cell accommodating both the gas samples and the custom QTF detector resulting in an increased compactness (2.5 cm of optical pathlength), suitable for real-time, in situ operation. The detection of C1 and C2 at different concentrations was selected as benchmark test, because of their pivotal role in the NG-related activities, from reservoirs analysis to pipeline leakages tracking, and the consequent demand for versatile sensors. Target analytes detection at percentage level was firstly investigated (C1 up to 10%, C2 up to 1% in N_2_), verifying the negligible influence of the surrounding medium on the QTF response when varying the gas matrix composition. Sensor calibration was then performed for both C1 and C2, separately, and the corresponding calibration curves were calculated by means of a monotonic, nonlinear fit with a Lambert-Beer-like function. The univariate calibrations were used to easily retrieve the analytes concentrations from C1-C2 binary mixtures, with an excellent accuracy as high as 98.4%, without involving multivariate analysis relying on extended spectra [27] rather than peak values. The ultimate sensor sensitivity was investigated by optimizing the beam spot position on the QTF surface, thus maximizing the detection performances. A linear response was observed at low concentrations for both the analytes, returning MDLs of ∼650 ppb and ∼90 ppb for C1 and C2 respectively at 10 s integration time. The achieved results demonstrated the possibility to operate the LITES sensor on target concentrations over six orders of magnitude, thus opening new possibilities for super-compact gas sensors based on direct absorption and low-cost light detectors, potentially operable with laser wavelengths covering the near- and mid-IR spectral region [33]. One possible future development will consist in testing this technology outside the laboratory, with the goal of exploiting the characteristics of LITES sensors on unmanned aerial vehicles (UAVs) for an efficient monitoring of NG supply chain [52].

## Declaration of Competing Interest

The authors declare the following financial interests/personal relationships which may be considered as potential competing interests: Angelo Sampaolo reports equipment, drugs, or supplies and travel were provided by Polytechnic University of Bari.

## Data Availability

Data will be made available on request.

## References

[1] Coulby G., Clear A., Jones O., Godfrey A. (2020). A scoping review of technological approaches to environmental monitoring. Int. J. Environ. Res. Public Health.

[2] Hayat H., Griffiths T., Brennan D., Lewis R.P., Barclay M., Weirman C., Philip B., Searle J.R. (2019). The state-of-the-art of sensors and environmental monitoring technologies in buildings. Sens. (Switz.).

[3] Selvaraj R., Vasa N.J., Nagendra S.M.S., Mizaikoff B. (2020). Advances in mid-infrared spectroscopy-based sensing techniques for exhaled breath diagnostics. Molecules.

[4] Feng C., Giglio M., Li B., Sampaolo A., Patimisco P., Spagnolo V., Dong L., Wu H. (2022). Detection of hydrogen sulfide in sewer using an erbium-doped fiber amplified diode laser and a gold-plated photoacoustic cell. Molecules.

[5] Moore C.W., Zielinska B., Pétron G., Jackson R.B. (2014). Air impacts of increased natural gas acquisition, processing, and use: a critical review. Environ. Sci. Technol..

[6] Speight J.G. (2018).

[7] Faramawy S., Zaki T., Sakr A.A.E. (2016). Natural gas origin, composition, and processing: a review. J. Nat. Gas. Sci. Eng..

[8] Ogden J., Jaffe A.M., Scheitrum D., McDonald Z., Miller M. (2018). Natural gas as a bridge to hydrogen transportation fuel: Insights from the literature. Energy Policy.

[9] A. Rojey and C. Jaffret, Natural Gas: Production, Processing, Transport (1997).

[10] Kuppusamy S., Maddela N.R., Megharaj M., Venkateswarlu K. (2020). Total Petroleum Hydrocarbons.

[11] Weller Z.D., Hamburg S.P., Von Fischer J.C. (2020). A national estimate of methane leakage from pipeline mains in natural gas local distribution systems. Environ. Sci. Technol..

[12] Chen C., Ren Q., Wang Y.Z. (2019). Review on multi gas detector using infrared spectral absorption technology. Appl. Spectrosc. Rev..

[13] Mchale L.E., Martinez B., Miller T.W., Yalin A.P. (2019). Open-path cavity ring-down methane sensor for mobile monitoring of natural gas emissions. Opt. Express.

[14] Defratyka S.M., Paris J.D., Yver-Kwok C., Loeb D., France J., Helmore J., Yarrow N., Gros V., Bousquet P. (2021). Ethane measurement by Picarro CRDS G2201-i in laboratory and field conditions: potential and limitations. Atmos. Meas. Tech..

[15] Ye W., Tu Z., Xiao X., Simeone A., Yan J., Wu T., Wu F., Zheng C., Tittel F.K. (2020). A NDIR mid-infrared methane sensor with a compact pentahedron gas-cell. Sensors 2020.

[16] Mahbub P., Noori A., Parry J.S., Davis J., Lucieer A., Macka M. (2020). Continuous and real-time indoor and outdoor methane sensing with portable optical sensor using rapidly pulsed IR LEDs. Talanta.

[17] Gong Z., Gao T., Mei L., Chen K., Chen Y., Zhang B., Peng W., Yu Q. (2021). Ppb-level detection of methane based on an optimized T-type photoacoustic cell and a NIR diode laser. Photoacoustics.

[18] Zifarelli A., Menduni G., Giglio M., Elefante A., Sukhinets A., Sampaolo A., Patimisco P., Fangyuan S., Chongwu W., Wang Q.J., Spagnolo V. (2022). Compact and versatile QEPAS-based sensor box for simultaneous detection of methane and infrared absorber gas molecules in ambient air. Front. Environ. Chem..

[19] Wu G., Gong Z., Li H., Ma J., Chen K., Peng W., Yu Q., Mei L. (2022). High-sensitivity multitrace gas simultaneous detection based on an all-optical miniaturized photoacoustic sensor. Anal. Chem..

[20] Leahu G., Li Voti R., Paoloni S. (2013). Trace gas analysis from glazes by means of a compact photothermal deflection spectroscopy apparatus. Rev. Sci. Instrum..

[21] Bertolotti M., Li Voti R. (2020). A note on the history of photoacoustic, thermal lensing, and photothermal deflection techniques. J. Appl. Phys..

[22] Li C., Dong L., Zheng C., Tittel F.K. (2016). Compact TDLAS based optical sensor for ppb-level ethane detection by use of a 3.34 μm room-temperature CW interband cascade laser. Sens. Actuators B Chem..

[23] He H., Gao S., Hu J., Zhang T., Wu T., Qiu Z., Zhang C., Sun Y., He S. (2021). In-situ testing of methane emissions from landfills using laser absorption spectroscopy. Appl. Sci..

[24] Barbosa M.F., Santos J.R.B., Silva A.N., Soares S.F.C., Araujo M.C.U. (2021). A cheap handheld NIR spectrometric system for automatic determination of methane, ethane, and propane in natural gas and biogas. Microchem. J..

[25] Ponte S., Andrade J.M., Vázquez C., Ferreiro B., Cobas C., Pérez A., Rey M., Vales C., Pellitero J., Santacruz B., Muniategui S., López-Mahía P., Shu B., Bettin H., Klaus D., Anders B., Betz M., Kühne U., Meier C., Eilts P. (2021). Modeling the natural gas knocking behaviour using gas-phase infrared spectra and multivariate calibration. J. Nat. Gas. Sci. Eng..

[26] Ferreiro B., Andrade J., López-Mahía P., Muniategui S., Vázquez C., Pérez A., Rey M., Vales C. (2021). Fast quality control of natural gas for commercial supply and transport utilities. Fuel.

[27] Menduni G., Zifarelli A., Sampaolo A., Patimisco P., Giglio M., Amoroso N., Wu H., Dong L., Bellotti R., Spagnolo V. (2022). High-concentration methane and ethane QEPAS detection employing partial least squares regression to filter out energy relaxation dependence on gas matrix composition. Photoacoustics.

[28] Sampaolo A., Patimisco P., Giglio M., Zifarelli A., Wu H., Dong L., Spagnolo V. (2021). Quartz-enhanced photoacoustic spectroscopy for multi-gas detection: a review. Anal. Chim. Acta.

[29] Olivieri A.C. (2018).

[30] He Y., Ma Y., Tong Y., Yu X., Tittel F.K. (2019). Ultra-high sensitive light-induced thermoelastic spectroscopy sensor with a high Q-factor quartz tuning fork and a multipass cell. Opt. Lett..

[31] Li J., Yu B., Zhao W., Chen W. (2014). A review of signal enhancement and noise reduction techniques for tunable diode laser absorption spectroscopy. Appl. Spectrosc. Rev..

[32] Dello Russo S., Zifarelli A., Patimisco P., Sampaolo A., Wei T., Wu H., Dong L., Spagnolo V. (2020). Light-induced thermo-elastic effect in quartz tuning forks exploited as a photodetector in gas absorption spectroscopy. Opt. Express.

[33] Wei T., Zifarelli A., Dello Russo S., Wu H., Menduni G., Patimisco P., Sampaolo A., Spagnolo V., Dong L. (2021). High and flat spectral responsivity of quartz tuning fork used as infrared photodetector in tunable diode laser spectroscopy. Appl. Phys. Rev..

[34] Ma Y., He Y., Patimisco P., Sampaolo A., Qiao S., Yu X., Tittel F.K., Spagnolo V. (2020). Ultra-high sensitive trace gas detection based on light-induced thermoelastic spectroscopy and a custom quartz tuning fork. Appl. Phys. Lett..

[35] Ma Y., Feng W., Qiao S., Zhao Z., Gao S., Wang Y., Wang Y. (2022). Hollow-core anti-resonant fiber based light-induced thermoelastic spectroscopy for gas sensing. Opt. Express.

[36] Mi Y., Ma Y. (2021). Ultra-highly sensitive ammonia detection based on light-induced thermoelastic spectroscopy. Sensors 2021.

[37] Liu X., Qiao S., Ma Y. (2022). Highly sensitive methane detection based on light-induced thermoelastic spectroscopy with a 2.33 µm diode laser and adaptive Savitzky-Golay filtering. Opt. Express.

[38] Zhang Q., Gong W., Chang J., Wei Y., Zhang T., Wang Z., Li Y., Zhang W., Liu T. (2022). Long-distance free space gas detection system based on QEPTS technique for CH4 leakage monitoring. Infrared Phys. Technol..

[39] Yacovitch T.I., Herndon S.C., Roscioli J.R., Floerchinger C., McGovern R.M., Agnese M., Pétron G., Kofler J., Sweeney C., Karion A., Conley S.A., Kort E.A., Nähle L., Fischer M., Hildebrandt L., Koeth J., McManus J.B., Nelson D.D., Zahniser M.S., Kolb C.E. (2014). Demonstration of an ethane spectrometer for methane source identification. Environ. Sci. Technol..

[40] Luo Z., Hao Q., Wang T., Li R., Cheng F., Deng J. (2020). Experimental study on the deflagration characteristics of methane-ethane mixtures in a closed duct. Fuel.

[41] Sampaolo A., Menduni G., Patimisco P., Giglio M., Passaro V.M.N., Dong L., Wu H., Tittel F.K., Spagnolo V. (2020). Quartz-enhanced photoacoustic spectroscopy for hydrocarbon trace gas detection and petroleum exploration. Fuel.

[42] Menduni G., Sampaolo A., Patimisco P., Giglio M., Dello Russo S., Zifarelli A., Elefante A., Wieczorek P.Z., Starecki T., Passaro V.M.N., Tittel F.K., Spagnolo V. (2020). Front-end amplifiers for tuning forks in quartz enhanced photoacoustic spectroscopy. Appl. Sci..

[43] Giglio M., Menduni G., Patimisco P., Sampaolo A., Elefante A., Passaro V.M.N., Spagnolo V. (2019). Damping mechanisms of piezoelectric quartz tuning forks employed in photoacoustic spectroscopy for trace gas sensing. Phys. Status Solidi Appl. Mater. Sci..

[44] Hosaka H., Itao K., Kuroda S. (1995). Damping characteristics of beam-shaped micro-oscillators. Sens. Actuators A Phys..

[45] Guo B. (2019). Well Productivity Handbook.

[46] Patimisco P., Sampaolo A., Dong L., Giglio M., Scamarcio G., Tittel F.K., Spagnolo V. (2016). Analysis of the electro-elastic properties of custom quartz tuning forks for optoacoustic gas sensing. Sens. Actuators B Chem..

[47] Sader J.E. (1998). Frequency response of cantilever beams immersed in viscous fluids with applications to the atomic force microscope. J. Appl. Phys..

[48] NIST, Chemistry WebBook (2022), SRD 69.

[49] Downs C., Vandervelde T.E. (2013). Progress in infrared photodetectors since 2000. Sens. (Switz.).

[50] Gordon I.E., Rothman L.S., Hargreaves R.J., Hashemi R., Karlovets E.V., Skinner F.M., Conway E.K., Hill C., Kochanov R.V., Tan Y., Wcisło P., Finenko A.A., Nelson K., Bernath P.F., Birk M., Boudon V., Campargue A., Chance K.V., Coustenis A., Drouin B.J., Flaud J.M., Gamache R.R., Hodges J.T., Jacquemart D., Mlawer E.J., Nikitin A.V., Perevalov V.I., Rotger M., Tennyson J., Toon G.C., Tran H., Tyuterev V.G., Adkins E.M., Baker A., Barbe A., Canè E., Császár A.G., Dudaryonok A., Egorov O., Fleisher A.J., Fleurbaey H., Foltynowicz A., Furtenbacher T., Harrison J.J., Hartmann J.M., Horneman V.M., Huang X., Karman T., Karns J., Kassi S., Kleiner I., Kofman V., Kwabia-Tchana F., Lavrentieva N.N., Lee T.J., Long D.A., Lukashevskaya A.A., Lyulin O.M., Makhnev V.Y., Matt W., Massie S.T., Melosso M., Mikhailenko S.N., Mondelain D., Müller H.S.P., Naumenko O.V., Perrin A., Polyansky O.L., Raddaoui E., Raston P.L., Reed Z.D., Rey M., Richard C., Tóbiás R., Sadiek I., Schwenke D.W., Starikova E., Sung K., Tamassia F., Tashkun S.A., Vander Auwera J., Vasilenko I.A., Vigasin A.A., Villanueva G.L., Vispoel B., Wagner G., Yachmenev A., Yurchenko S.N. (2022). The HITRAN2020 molecular spectroscopic database. J. Quant. Spectrosc. Radiat. Transf..

[51] B.A. Ulrich, M. Mitton, E. Lachenmeyer, A. Hecobian, D. Zimmerle, and K.M. Smits, Natural Gas Emissions from Underground Pipelines and Implications for Leak Detection, (2019).

[52] Asadzadeh S., de Oliveira W.J., de Souza Filho C.R. (2022). UAV-based remote sensing for the petroleum industry and environmental monitoring: State-of-the-art and perspectives. J. Pet. Sci. Eng..

